# The first established microsatellite markers to distinguish *Candida orthopsilosis* isolates and detection of a nosocomial outbreak in China

**DOI:** 10.1128/jcm.00806-23

**Published:** 2023-10-25

**Authors:** Zhengyu Luo, Yating Ning, Shuying Yu, Meng Xiao, Rongchen Dai, Xinfei Chen, Yao Wang, Wei Kang, Yan Jiang, Hua Yu, Hongjie Liang, Yingchun Xu, Tianshu Sun, Li Zhang

**Affiliations:** 1 Department of Laboratory Medicine, State Key Laboratory of Complex, Severe, and Rare Diseases, Peking Union Medical College Hospital, Chinese Academy of Medical Sciences and Peking Union Medical College, Beijing, China; 2 Graduate School, Chinese Academy of Medical Sciences and Peking Union Medical College, Beijing, China; 3 Beijing Key Laboratory for Mechanisms Research and Precision Diagnosis of Invasive Fungal Diseases, Beijing, China; 4 Department of Microbiology and Immunology, Guizhou Medical University Affiliated Hospital, Guiyang, China; 5 Department of Laboratory Medicine and Sichuan Provincial Key Laboratory for Human Disease Gene Study, Sichuan Provincial People’s Hospital, University of Electronic Science and Technology of China, Chengdu, China; 6 Department of Clinical Laboratory, Key Laboratory of Clinical Laboratory Medicine of Guangxi Department of Education, The First Affiliated Hospital of Guangxi Medical University, Guangxi, China; 7 Clinical Biobank, Medical Research Center, National Science and Technology Key Infrastructure on Translational Medicine, Peking Union Medical College Hospital, Chinese Academy of Medical Science and Peking Union Medical College, Beijing, China; University of Utah, Salt Lake City, Utah, USA

**Keywords:** *Candida orthopsilosis*, microsatellite typing, microsatellite loci, outbreak, AFLP

## Abstract

The infection proportion of *Candida orthopsilosis*, a member of the *C. parapsilosis* complex, has increased globally in recent years, and nosocomial outbreaks have been reported in several countries. This study aimed to establish microsatellite loci-based typing method that was able to effectively distinguish among *C. orthopsilosis* isolates. Three reference *C. orthopsilosis* genome sequences were analyzed to identify repeat loci. DNA sequences containing over eight bi- or more nucleotide repeats were selected. A total of 51 loci were initially identified, and locus-specific primers were designed and tested with 20 epidemiologically unrelated isolates. Four loci with excellent reproducibility, specificity, and resolution for molecular typing purposes were identified, and the combined discriminatory power (DP, based on 20 epidemiologically unrelated isolates) of these four loci was 1.0. Reproducibility was demonstrated by consistently testing three strains each in triplicate, and stability, demonstrated by testing 10 successive passages. Then, we collected 48 *C*. *orthopsilosis* non-duplicate clinical isolates from the China Hospital Invasive Fungal Surveillance Net study to compare the DP of the microsatellite-based typing with internal transcribed spacer (ITS) and amplified fragment length polymorphism (AFLP) typing analyses, using ATCC 96139 as a reference strain. These 49 isolates were subdivided into 12 microsatellite types (COMT1–12), six AFLP types, and three ITS types, while all the isolates with the same COMT belonged to consistent AFLP and ITS type, demonstrating the high DP of our microsatellite-type method. According to our results, COMT12 was found to be the predominant type in China, and COMT5 was the second largest and responsible for causing a nosocomial outbreak. This microsatellite-type method is a valuable tool for the differentiation of *C. orthopsilosis* and could be vital for epidemiological studies to determine strain relatedness and monitor transmission.

## INTRODUCTION

*Candida albicans* is responsible for the vast majority of invasive fungal infections; however, in recent years, the proportion of infections caused by non-*albicans Candida* spp., such as *C. parapsilosis*, *C. auris*, *C. glabrata*, and *C. tropicalis*, has steadily increased worldwide (1). Of note, *C. parapsilosis* complex has become the second-to-third most common causative agent of invasive infections, accounting for more than 20% of *Candida* bloodstream infections in Brazil, Argentina, Spain, and China and more than 30% in Turkey, Greece, Romania, and South Africa (2 – 4). The *C. parapsilosis* complex has been shown to invade adult patients in intensive care units as well as newborns (5, 6), and it can colonize the hands of healthcare workers and develop recalcitrant biofilms on medical apparatus and instruments such as central venous catheter, leading to nosocomial spread (7).

**Fig 1:**
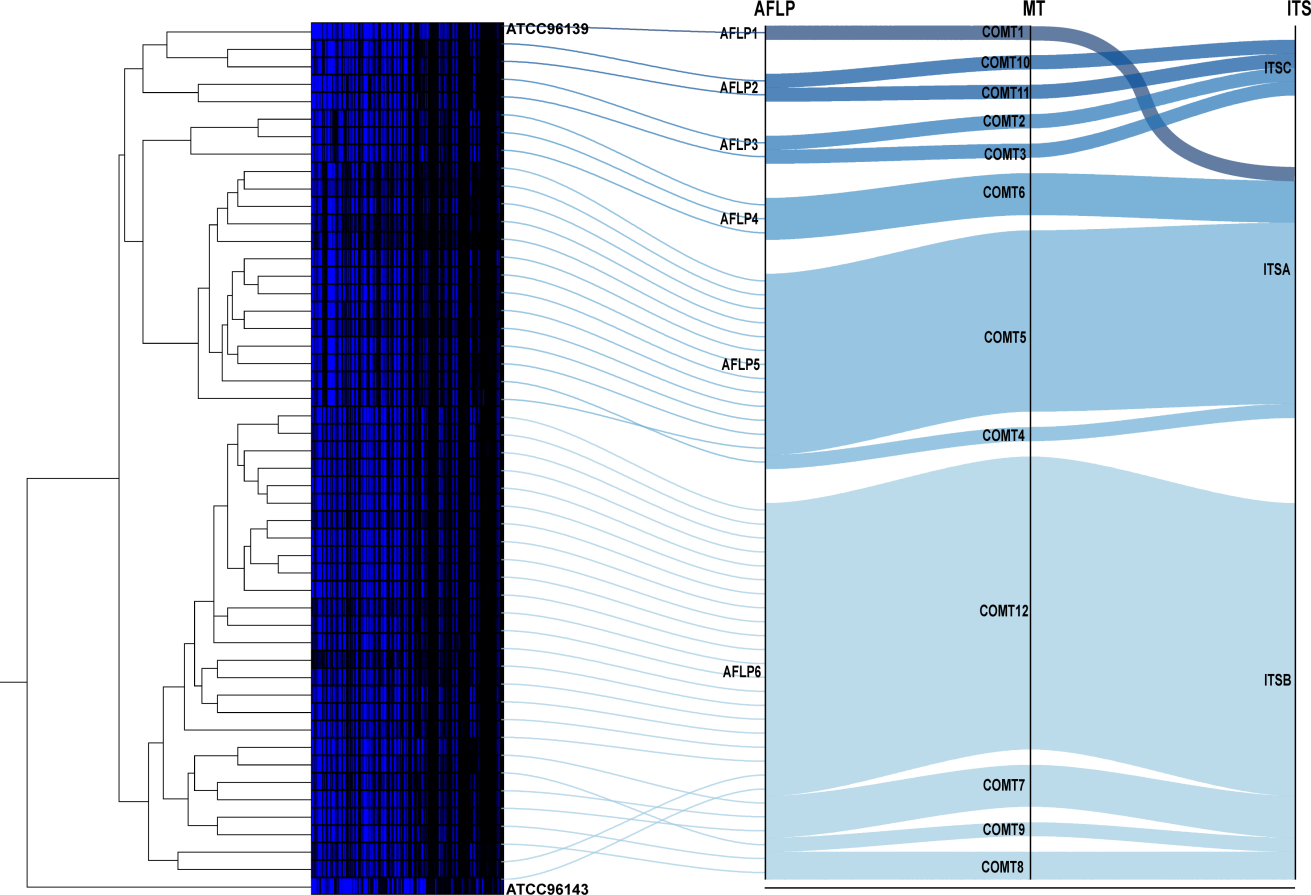
AFLP analysis of 48 isolates and the reference strain ATCC 96139 and the corresponding relationships among AFLP, COMT, and ITS classification methods. The AFLP analyses results are presented on the left side of the graph, with the thin lines corresponding to AFLP types. The bold lines show the corresponding relationship between AFLP types and MT types, as well as MT types and ITS types. The colors of the bold lines only indicate different AFLP types.

Phylogenetic analyses have divided the *C. parapsilosis* complex into three separate clusters: *C. parapsilosis sensu stricto*, *C. orthopsilosis*, and *C. metapsilosis* (8, 9). The isolation rate of *C. orthopsilosis* has steadily increased from 0.4% (2009–2014) to 1.8% (2015–2017) of all *Candida* spp. according to the China Hospital Invasive Fungal Surveillance Net (CHIF-NET) study (10). Specifically, the annual isolation rate was 0.5% in 2015, 2.3% in 2016, and 2.6% in 2017, indicating that the expansion of this species is an emergent healthcare issue. More alarming is the emergence of horizontal transmission of *C. orthopsilosis*, as demonstrated by a multicenter study in Iran that found that four blood-derived isolates from different patients clustered into two genotypes with high similarities (11). Over the last two decades, several incidents of *C. orthopsilosis* outbreaks have occurred (12, 13), including a small nosocomial outbreak in a hospital in Brazil (13). In these cases, the patients infected with *C. orthopsilosis* had poor prognoses (7, 14) (Fig. 1).

Strain typing methods are essential for molecular epidemiological studies and outbreak surveillance. Several strain typing methods, including random amplification of polymorphic DNA analysis (13), internal transcribed spacer (ITS) sequencing (15), and restriction fragment length polymorphism analysis (16), have been used to discriminate members of the *C. parapsilosis* complex. Multilocus sequence typing (MLST) is the gold standard for typing both fungi and bacteria (17, 18), and MLST methods have now been established for *C. albicans*, *C. dubliniensis*, *C. glabrata*, *Issatchenkia orientalis*, and *C. tropicalis* (19). Nevertheless, MLST exhibits limited resolution when applied to *C. parapsilosis*, and a comprehensive MLST scheme specific to this species has not yet been defined, which limits its clinical usage (12). Amplified fragment length polymorphism (AFLP) has traditionally served as a widely employed typing method for the *C. parapsilosis* complex, as well as *Candida* spp. (12, 20 – 22). However, its practical application presents several challenges, including the need for high-quality templates and specialized operators. Furthermore, AFLP lacks feasibility for inter-hospital comparisons, making its adoption at the grassroots level a significant challenge.

Currently, the microsatellite-based typing method demonstrates superior discriminatory power (DP) compared to AFLP and could distinguish between isolates with low degrees of sequence variation (23); therefore, it has been extensively utilized for the identification and investigation of fungal outbreaks (24, 25). Microsatellite typing is a rapid molecular method used for analyses of genetic variation in *Candida* species via the development and highly accurate identification of short tandem repeat (STR) polymorphisms (26, 27), and it has been utilized in genotyping analyses of *C. albicans*, *C. glabrata*, *C. parapsilosis sensu stricto*, and *C. tropicalis* (24, 28, 29). For example, Sabino et al. (30) developed four microsatellite markers to genotype *C. parapsilosis sensu stricto* with a high DP, and Zhang et al. (31) used these four markers to identify 122 microsatellite types in 319 isolates. These studies demonstrated that microsatellite typing is applicable to both epidemiology studies and outbreak surveillance of *C. parapsilosis sensu stricto*.

Despite the applications of microsatellite typing in identifying various *Candida* spp., it has not yet been applied to the typing of *C. orthopsilosis*. Therefore, in this study, we collected *C. orthopsilosis* isolates from the CHIF-NET study and developed a microsatellite-based typing method using a set of markers for molecular typing and genetic variation analysis of *C. orthopsilosis*. The results of this study will be useful for epidemiological purpose to determine strain relatedness and monitor transmission.

## MATERIALS AND METHODS

### Isolates selection and identification

A total of 68 non-duplicate *C. orthopsilosis* isolates from the CHIF-NET study, along with the reference strain ATCC 96139, were utilized in this study for validating the selected microsatellite loci. All strains were identified down to the subspecies level by analyzing the sequences of the ITS region (21, 32). Yeast cells of *C. orthopsilosis* were grown on yeast extract peptone dextrose medium at 30°C overnight prior to DNA extraction, which was performed using the Fungi Genomic DNA Extraction Kit (Solarbio Science & Technology, Beijing, China) according to the company’s recommended protocols.

### Screening and selection of repeat loci in a *C. orthopsilosis* genome sequence database

*C. orthopsilosis* whole reference genome sequences were obtained from the GenBank database (MCO456: GCA_900002835.2; AY2: GCA_000304155.1; and Co90-125: GCA_000315875.1). The RepeatMasker software was used to select the repeat loci that were shared between the reference genomes (33). According to previously published reports (24, 30), we chose DNA sequences that contained more than eight bi- or more nucleotide repeats to yield sequences with greater genetic variability. The Primer3 software was then used to design forward and reverse primers to amplify the loci that met the criteria above. Gradient PCR was performed to optimize the primer annealing temperatures, and PCR was performed to amplify the DNA sequences using conditions described previously (34).

### Polyacrylamide gel electrophoresis

PCR amplification products were separated by electrophoresis on 6% denaturing polyacrylamide gels (0.5 mM thick) in Tris-borate EDTA electrophoresis buffer. An equal volume of loading buffer (98% formamide, 10 mmol/L EDTA, 0.25% xylene cyanin, and 0.25% bromophenol blue) was added to the selective amplification products prior to denaturation at 95℃ for 5 min. Following the loading of samples (8 µL) onto the gel, power was applied at a constant 100 W for 2 min then adjusted to 60 W at 43℃. Electrophoresis was considered complete when the xylene blue line migrated approximately 2/3 of the way down the gel. After electrophoresis, DNA bands were visualized by silver staining.

### Microsatellite-based typing

Each PCR mix (20 µL) contained 2 µL of DNA template, 10 µL of mix buffer, 7.4 µL of ddH_2_O, and 20 µM of forward and reverse primers. PCR amplifications were performed with a total of 27 cycles under the following conditions: denaturation at 94°C for 30 s, annealing at 60°C for 40 s, and extension at 72°C for 50 s, followed by a final extension step of 10 min at 72°C. The forward primers were labeled with 6-carboxyfluorescein (FAM). A mixture of 15 µL of formamide and internal size marker at a volume ratio of 100:1 was added to the upper plate, followed by 1 µL of 10-fold diluted PCR product. Capillary electrophoresis (CE) was performed with an ABI 3730XL DNA Analyzer (Applied Biosystems, Foster City, CA, USA). Raw data were analyzed by using Fragment (Plant) analyses software in Genemarker (Version 1.51). The position of the internal size marker in each lane was compared and analyzed with the position of the peak value of each sample to obtain the fragment size. Bionumerics (Version 7.6) was used to perform data analyses. Isolates were categorized as the same microsatellite type only if they exhibited exactly the same fragment size at all four microsatellite loci. A cluster was defined as the microsatellite type containing at least two isolates with identical allelic profiles (35).

### AFLP analysis

AFLP analysis was carried out by mixing approximately 50 ng of genomic DNA with restriction ligation containing EcoRI and MseI restriction enzymes and complementary adaptors as described previously (36). Tris-HCl (pH 8.3) buffer was added to dilute the pre-amplified product 50-fold, and 5 µL of the diluted product was subjected to amplification reaction mixture containing the selective primers EcoRI (5′-FAM-GACTGCGTACCAATTCAC-3′) and MseI (5′-GATGAGTCCTGACTAAC-3′), 2× PCR mix 10 µL, Eprimer (1 µL, final concentration 20 µM), Mprimer (1 µL, final concentration 20 µM), and ddH_2_O. An aliquot (1 µL) of the 10-fold diluted solutions of PCR amplified products was added to a mixture of 8.9 µL of water and 0.1 µL of internal size marker, followed by fragment analyses on an ABI 3500xL Genetic Analyzer according to the instructions of the manufacturer (Applied Biosystems).

### Reproducibility and stability

The reproducibility of the microsatellite typing method was assessed three times by using the DNA preparations obtained from the same strain selected randomly, and the stability was assessed following 10 successive cultures on Sabouraud agar medium; additionally, the reference strain ATCC 96139 was included as a control in each run.

### Statistical analysis

Allelic and genotypic frequencies were determined by using ARLEQUIN (version 3.5.2.2) software. The DP was calculated using the Simpson index as follows (37): $DP=1-\frac{1}{N(N-1)}\sum_{j-1}^{S}nj(nj-1)$ , where *N* is the number of strains, *S* is the total number of different genotypes, and *nj* is the number of strains of genotype *j*.

## RESULTS

### Screening of repeat loci in the *C. orthopsilosis* genome

Three reference genome sequences from the standard strains MCO456, AY2, and Co90-125 were obtained from the *C. orthopsilosis* genome sequence database to screen for repeat loci. Based on the DNA sequences of the ITS regions, MCO456 and AY2 were both found to be of the ITS-A type, while Co90-125 was found to be of a non-ITS-A/B/C type. Average nucleotide identity analysis (38) demonstrated that MCO456 and AY2 exhibited high homology, and there were intraspecific differences among MCO456, AY2, and Co90-125, which supports the credibility of the sequencing data. RepeatMasker software (Version 4.0.5) was used to select loci from each genome of the three reference sequences, in which 2,265 loci in MCO456, 2,152 loci in AY2, and 1,881 loci in Co90-125 were identified; a total of 300 loci were shared by all the three genomes. Among the 300 common loci, 51 were found to contain at least eight bi- or more nucleotide repeats, so these loci were chosen for further locus-specific primer design.

### Selection of high-resolution microsatellite markers

To evaluate the resolution of the method and identification of the polymorphisms, the PCR amplification products were separated by PAGE to analyze the loci in each isolate from the different origins. The markers chosen for these analyses were found to be species specific, as no amplification products were found in sibling species, *C. parapsilosis sensu stricto*, *C. metapsilosis*, and *Lodderomyces elongisporus*, as well as other common pathogenic fungi, including *C. albicans*, *Issatchenkia orientalis*, *C. tropicalis*, and *C. glabrata*. The nomenclature chosen for the new markers was CO, named after *C. orthopsilosis*, followed by a number denoting the order in which they were studied in the experiments. Of the original 51 loci, six loci were found to have excellent specificity and stability: CO4, CO8, CO11, CO32, CO49, and CO51.

These six loci were used to type 20 epidemiologically unrelated *C. orthopsilosis* isolates from 16 hospitals in 12 provinces across China. PCR was performed using fluorescently labeled primers, and the products were analyzed by CE. According to the results, CO4 identified six microsatellite types in 20 isolates with the highest DP value (0.85), CO11 identified six microsatellite types, CO49 identified five types, and CO32 identified four types; the CO11, CO49, and CO32 loci demonstrated a DP value of 0.83. Both CO8 and CO51 identified three microsatellite types, with the lowest DP value of 0.62. We ultimately selected four loci (CO4, CO11, CO32, and CO49) that could classify these 20 isolates into 20 different microsatellite types with a DP of 1.0 (Table 1). The analysis of the 20 isolates revealed that all four microsatellite loci selected were polymorphic, presenting four to five alleles and from four to six different microsatellite types. The size ranges of the alleles as well as other detailed information are shown in Table 2.

**TABLE 1:** Microsatellite DNA sequences selected, copy number, and primers used for PCR amplification

STR	Repetitive motif	Primer sequence
CO4	(TCT)_12_	F: FAM-TGTTGTCTGAAATACAGTCCAATTGA
		R: ATGTTGCACCGAAACTTACG
CO11	(TTG)_12_	F: FAM-CGGTTGTTTTAGTGGCAGCA
		R: CGAGCCTATTCAAGTACTGTTGA
CO32	(TC)_15_	F: FAM-ATCCGCTGTGACTCCTCTCT
		R: ACCAGCCATCTTTGGACTTT
CO49	(CAA)_8_	F: FAM-AGTCATCATTTACCGCCGCA
		R: ACCAGCAGGATTATTATTAGCAGC

**TABLE 2:** Characteristics of the microsatellite loci selected

STR	Size range (bp)	No. of alleles	Allele frequency	No. of genotypes	Genotype frequency	DP	%Heterozygosity
CO4	249–282	5	0.1–0.3	6	0.1–0.3	0.85	15
CO11	263–301	5	0.05–0.375	6	0.05–0.35	0.83	15
CO32	249–273	4	0.05–0.65	4	0.1–0.4	0.83	60
CO49	240–267	5	0.05–0.55	5	0.1–0.4	0.83	40

### Reproducibility and stability of the four microsatellite loci

The microsatellite types remained unchanged when analyzing the DNA from the same strain within the same run or across different runs. In addition, for all the generations tested, the genotypes remained consistent with their original generation. This finding further reinforced the robustness and accuracy of our microsatellite typing method for *C. orthopsilosis*.

### Verification of the four microsatellite loci and comparison of ITS sequencing and AFLP typing results

Forty-eight non-duplicate *C. orthopsilosis* isolates, collected by the CHIF-NET 2017–2019 study from 23 hospitals in 15 provinces, and the reference strain ATCC 96139 underwent by microsatellite typing, ITS sequencing, and AFLP analyses to compare the DP of the four microsatellite loci. Forty-nine strains were subdivided into 12 microsatellite types (COMT), while into six AFLP types and three ITS types (Fig. 1; Table S1).

ITS-A included 18 strains in total, which were classified as AFLP1, AFLP4, and AFLP5 types. The ITS-A type strain ATCC 96139 was identified as COMT1 and AFLP1 type. Strains of the AFLP4 type (*n* = 3) were all classified as the COMT6 type. Strains of the AFLP5 type (*n* = 14) included 1 COMT4 strain and 13 COMT5 strains. All ITS-B strains, 27 in total, were of the AFLP6 type, and they were classified as four different COMT types: COMT7 (*n* = 3), COMT8 (*n* = 2), COMT9 (*n* = 1), and COMT12 (*n* = 21). All ITS-C stains, four in total, included strains that were classified as AFLP2 and AFLP3 types. Here, the AFLP2 type included strains belonging to the COMT10 (*n* = 1) and COMT11 (*n* = 1) types, and the AFLP3 type included strains belonging to the COMT2 (*n* = 1) and COMT3 (*n* = 1) types (Fig. 1). Accordantly, all the isolates with the same microsatellite type belonged to consistent AFLP type, as well as ITS type. These results indicated that these three methods were highly consistent, but our microsatellite-based typing method showed a higher DP.

### Potential nosocomial outbreaks in China

**Fig 2:**
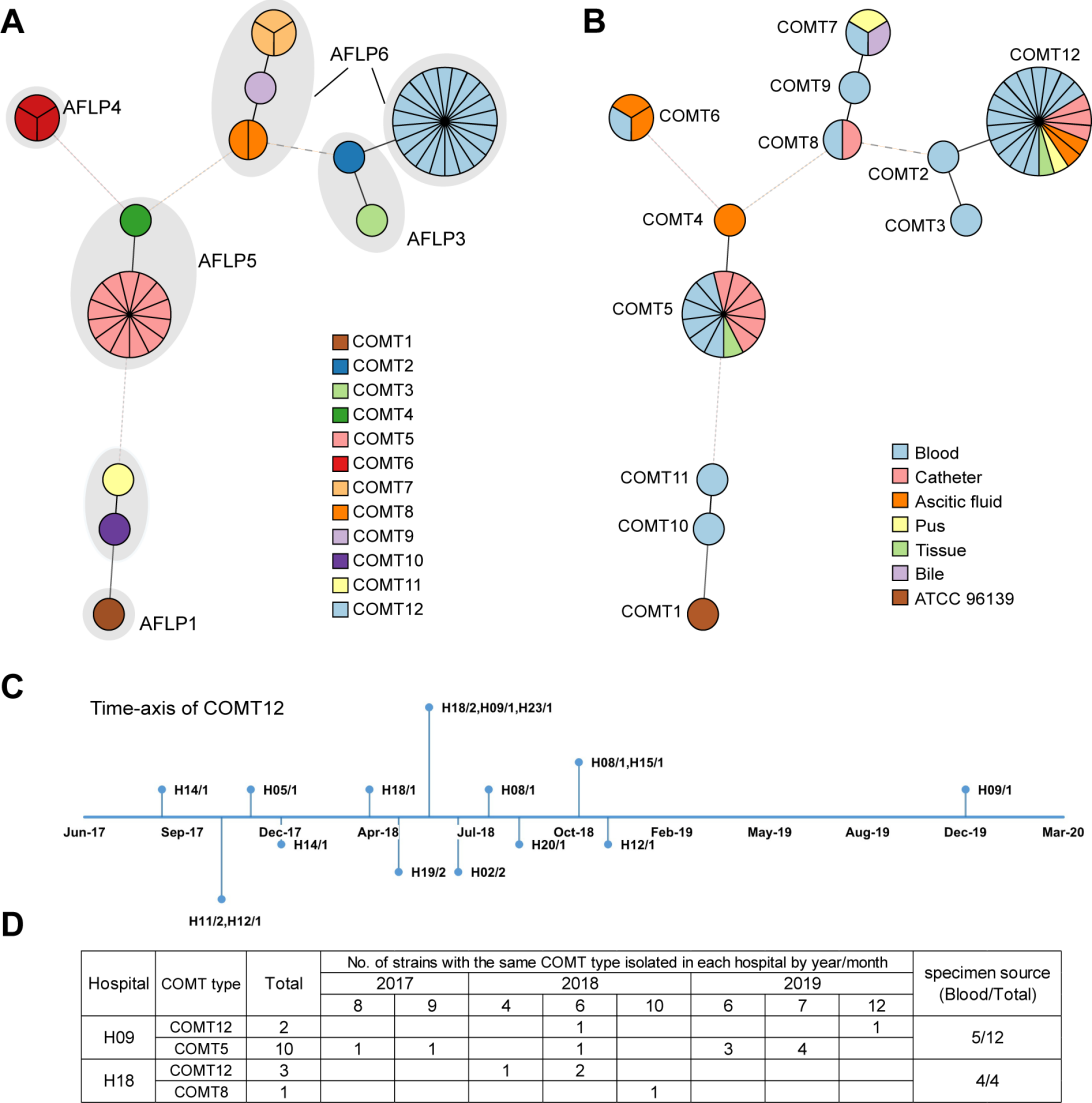
The microsatellite types and clinical characteristics of 48 invasive *C. orthopsilosis.* (**A**) Minimum spanning tree analysis of 49 *C*. *orthopsilosis* based on allelic profiles at four microsatellite loci, and the shadow indicates the AFLP type. (**B**) Minimum spanning tree analysis of 49 *C*. *orthopsilosis*, and the colors represent different sample types. (**C**) Time axis of COMT12 type isolates. Strains are indicated as the hospital of isolation and the isolate number. (**D**) Number of strains isolated in hospitals H09 and H18 by year and month.

Forty-eight invasive clinical isolates fell into 11 microsatellite types, 6 COMTs of which contained only one isolate and 5 COMTs (COMT12, COMT5, COMT6, COMT7, and COMT8) considered as belonging to clusters (Fig. 2A). COMT12 was the predominant type in China, and this cluster contained 21 isolates (37.5%) from 12 hospitals in nine different provinces. Among these, 47.6% (10/21) isolates were derived from blood samples. Three COMT12 isolates were found in hospital H18, where all patients had an invasive bloodstream infection, and two strains were isolated from different patients on the same day (Fig. 2B and C). The second largest cluster, COMT5, contained 13 isolates, 10 of which were found in hospital H09 and first appeared twice in August 2017, only once in 2018, and persisted until July 2019, when it reached maximum isolation rate. The 10 patients infected with the COMT5 type strain had mainly interacted with the general surgery department and cardiovascular surgical care unit. Among them, five isolates were cultured from blood, and the other five isolates were cultured from the vascular catheter (Fig. 2C and D). These findings suggested the presence of a potential outbreak in the surgical wards in H09, as most of the patients were infected within a short time span in June and July 2019.

## DISCUSSION

Recently, *C. parapsilosis* was classified as a fungal pathogen of high priority by the World Health Organization (39). The ability of *C. parapsilosis* to form biofilms enhances its ability to cause invasive infections that exhibit a mortality rate between 20% and 45%. *C. orthopsilosis*, one particular member of the *C. parapsilosis* complex, causes a wide range of clinical manifestations, ranging from superficial skin infections to fatal invasive bloodstream infections (11). Nosocomial outbreaks of *C. orthopsilosis* have been reported in multiple hospitals worldwide. Therefore, it is essential to develop accurate genotyping tools to distinguish *C. orthopsilosis* genotypes from other members of the *C. parapsilosis* complex. In addition, accurate *C. orthopsilosis* genotyping methods would lead to a deeper understanding of the genetic characteristics of this species and aid in further investigations of the epidemic isolates.

Based on DNA sequence data of the ITS region, we identified three haplotypes (ITS-A, ITS-B, and ITS-C) among 48 *C*. *orthopsilosis* isolates selected from CHIF-NET study 2017–2019 as well as a reference strain ATCC 96139. The ITS-B type was the most common type among the 49 isolates, while the ITS-C type was the least common, which was consistent with the results of previous studies (21, 40, 41). The reference genome strain Co90-125 did not match any ITS type, and no strain had an ITS sequence that was exactly consistent with Co90-125; this could be due to its origin as a hybridization event between *C. orthopsilosis* subspecies ITS-A and ITS-B (21).

AFLP is a molecular technique that can be used to produce a DNA fingerprint of an organism. AFLP has previously been used to type *C. orthopsilosis* strains. Tavanti et al. (12) used AFLP to identify 12 distinct genotypes from 33 *C*. *orthopsilosis* isolates obtained from 13 patients. Similarly, De Carolis et al. (22) used AFLP to identify six distinct genotypes among eight isolates from an individual patient. In our study, six AFLP types were detected in 49 isolates: AFLP1 (*n* = 1, the reference strain 96139), AFLP2 (*n* = 2), AFLP3 (*n* = 2), AFLP4 (*n* = 3), AFLP5 (*n* = 14), and AFLP6 (*n* = 27). Despite AFLP demonstrating a greater discriminatory ability compared to ITS, the isolates clustered within AFLP5 and AFLP6 could not be further divided by the AFLP method.

Microsatellite loci have considerable variations in length and high levels of polymorphism, which make them excellent markers for a number of different genetic analyses, including genotyping and population structure analyses. Furthermore, the results of such analyses have been shown to remain stable over many generations (42). Microsatellite typing methods have been successfully used to type a few *Candida* species and other fungi, corroborating the potential for these typing methods in clinical practice. Wu et al. (24) reported that some singletons in their MLST analyses formed new groups when analyzed using the microsatellite typing method, and they noted that such techniques could further divide the strains gathered by MLST into different groups. Huang et al. (34) hypothesized that *Rhodotorula mucilaginosa* might be able to persist in the hospital environment, since identical MT types were isolated in the same hospital in different years. These studies illustrate the potential of microsatellite typing as a tool for identifying and characterizing fungal strains in clinical settings.

To our best knowledge, this is the first report that developed and tested microsatellite markers for application to *C. orthopsilosis*. We demonstrated that four loci (CO4, CO11, CO32, and CO49) could effectively characterize selected *C. orthopsilosis* strains among 12 COMT types, and the results of the method were consistent with those AFLP and ITS typing. Notably, the COMT types matched the AFLP and ITS types precisely and could divide the strains clustered in the same AFLP or ITS types into different clusters. For instance, all isolates of the COMT5 type were associated with AFLP5 and ITS-A, while isolates of the COMT12 type were exclusively associated with AFLP6 and ITS-B. This high level of consistency further corroborated the reliability and accuracy of our approach. AFLP analyses divided the ITS-A group into AFLP4 and AFLP5 but were unable to further differentiate the ITS-B group into additional types. However, the microsatellite typing method proved to be highly effective in subdividing the AFLP5/ITS-B groups into four distinct and unique COMT types (COMT7, COMT8, COMT9, and COMT12). Moreover, within the ITS-C group, two isolates were associated with AFLP2, and two were associated with AFLP3, but the four isolates also belonged to four different COMT types (COMT10, COMT11, COMT2, and COMT3). These results demonstrated the powerful ability of microsatellite typing method to type *C. orthopsilosis* species and evaluate the extent of intraspecific genetic variation in *C. orthopsilosis*. Furthermore, the microsatellite typing method demonstrated outstanding reproducibility and specificity. The results remained consistent when DNA extracted from the same isolate was used, further validating the reliability of the method. Crucially, when attempting to type other *Candida* species, such as *C. albicans*, *Issatchenkia orientalis*, *C. tropicalis*, and *C. glabrata*, no amplification products were observed using the four loci, underscoring the specificity of the microsatellite typing method for *C. orthopsilosis*.

In a clinical setting, the microsatellite typing method was shown to help detect the potential COMT5 outbreak in hospital H09. A total of 10 *C. orthopsilosis* isolates were isolated from the blood and vascular catheters of patients in both the general surgery department and cardiovascular surgical care unit. While the prevalence of *C. orthopsilosis* may differ across countries, the isolation of *C. orthopsilosis* from blood samples has been previously reported (11, 43), highlighting its potential role in nosocomial bloodstream fungal infections. This was the first report of *C. orthopsilosis* outbreak in China, suggesting the possible existence of reservoirs of infection and necessitating the need for ongoing surveillance and infection control measures to prevent outbreaks. Overall, these findings underscore the importance of developing advanced molecular methods for epidemiological studies of *C. orthopsilosis* to provide invaluable insights into the transmission and diversity of *C. orthopsilosis*.

Despite the successful application of the microsatellite typing method in the characterization of *C. orthopsilosis* strains, this study was constrained by its retrospective design, resulting in the unavailability of certain clinical details. Furthermore, the inability to monitor medical workers’ hands, the facilities, or the environment surrounding patients prevented us from tracing transmission routes effectively. Additionally, the lack of isolates from other countries restricted our ability to analyze the variability of microsatellite types in a broader context.

In conclusion, we first developed new microsatellite loci for *C. orthopsilosis*. The four loci selected exhibited high DP, and they were shown to be more powerful than ITS sequencing and AFLP analyses. These microsatellite markers will be a valuable tool for distinguishing *C. orthopsilosis* for epidemiologically studies, investigating nosocomial cross-transmission, and other potential applications. While this method was operationally trivial and highly reproducible, more *C. orthopsilosis* isolates will need to be sequenced and analyzed to fully uncover the potential of these newly developed microsatellite markers, and optimization of the experimental procedures and standardization are necessary for maximum accuracy and sensitivity.

## SUPPLEMENTAL MATERIAL

Table S1 jcm.00806-23-s0001.docx The fragment size of four loci of 48 samples and ATCC96139.

ASM does not own the copyrights to Supplemental Material that may be linked to, or accessed through, an article. The authors have granted ASM a non-exclusive, world-wide license to publish the Supplemental Material files. Please contact the corresponding author directly for reuse.

## References

[1] Enoch DA , Yang H , Aliyu SH , Micallef C . 2017. The changing epidemiology of invasive fungal infections. Methods Mol Biol 1508:17–65. doi:10.1007/978-1-4939-6515-1_2 27837497

[2] Lamoth F , Lockhart SR , Berkow EL , Calandra T . 2018. Changes in the epidemiological landscape of invasive candidiasis. J Antimicrob Chemother 73:i4–i13. doi:10.1093/jac/dkx444 29304207 PMC11931512

[3] Tóth R , Nosek J , Mora-Montes HM , Gabaldon T , Bliss JM , Nosanchuk JD , Turner SA , Butler G , Vágvölgyi C , Gácser A . 2019. Candida parapsilosis: from genes to the bedside. Clin Microbiol Rev 32:e00111-18. doi:10.1128/CMR.00111-18 30814115 PMC6431126

[4] Daneshnia F , de Almeida Júnior JN , Ilkit M , Lombardi L , Perry AM , Gao M , Nobile CJ , Egger M , Perlin DS , Zhai B , Hohl TM , Gabaldón T , Colombo AL , Hoenigl M , Arastehfar A . 2023. Worldwide emergence of fluconazole-resistant Candida parapsilosis: current framework and future research roadmap. Lancet Microbe 4:e470–e480. doi:10.1016/S2666-5247(23)00067-8 37121240 PMC10634418

[5] Harrington R , Kindermann SL , Hou Q , Taylor RJ , Azie N , Horn DL . 2017. Candidemia and invasive candidiasis among hospitalized neonates and pediatric patients. Curr Med Res Opin 33:1803–1812. doi:10.1080/03007995.2017.1354824 28699797

[6] Zuo XS , Liu Y , Hu K . 2021. Epidemiology and risk factors of candidemia due to Candida parapsilosis in an intensive care unit. Rev Inst Med Trop Sao Paulo 63:e20. doi:10.1590/S1678-9946202163020 33787740 PMC7997672

[7] Trofa D , Gácser A , Nosanchuk JD . 2008. Candida parapsilosis, an emerging fungal pathogen. Clin Microbiol Rev 21:606–625. doi:10.1128/CMR.00013-08 18854483 PMC2570155

[8] Tavanti A , Davidson AD , Gow NAR , Maiden MCJ , Odds FC . 2005. Candida orthopsilosis and Candida metapsilosis spp. nov. to replace Candida parapsilosis groups II and III. J Clin Microbiol 43:284–292. doi:10.1128/JCM.43.1.284-292.2005 15634984 PMC540126

[9] Cantón E , Pemán J , Quindós G , Eraso E , Miranda-Zapico I , Álvarez M , Merino P , Campos-Herrero I , Marco F , de la Pedrosa EGG , Yagüe G , Guna R , Rubio C , Miranda C , Pazos C , Velasco D , FUNGEMYCA Study Group . 2011. Prospective multicenter study of the epidemiology, molecular identification, and antifungal susceptibility of Candida parapsilosis, Candida orthopsilosis, and Candida metapsilosis isolated from patients with candidemia. Antimicrob Agents Chemother 55:5590–5596. doi:10.1128/AAC.00466-11 21930869 PMC3232769

[10] Xiao M , Sun Z-Y , Kang M , Guo D-W , Liao K , Chen SC-A , Kong F , Fan X , Cheng J-W , Hou X , Zhou M-L , Li Y , Yu S-Y , Huang J-J , Wang H , Xu Y-C , China Hospital Invasive Fungal Surveillance Net (CHIF-NET) Study Group . 2018. Five-year national surveillance of invasive candidiasis: species distribution and azole susceptibility from the China hospital invasive fungal surveillance net (CHIF-NET) study. J Clin Microbiol 56:e00577-18. doi:10.1128/JCM.00577-18 29743305 PMC6018329

[11] Arastehfar A , Khodavaisy S , Daneshnia F , Najafzadeh MJ , Mahmoudi S , Charsizadeh A , Salehi MR , Zarrinfar H , Raeisabadi A , Dolatabadi S , Zare Shahrabadi Z , Zomorodian K , Pan W , Hagen F , Boekhout T . 2019. Molecular identification, genotypic diversity, antifungal susceptibility, and clinical outcomes of infections caused by clinically underrated yeasts, Candida orthopsilosis and Candida metapsilosis: an Iranian multicenter study. Front Cell Infect Microbiol 9:264. doi:10.3389/fcimb.2019.00264 31417877 PMC6682699

[12] Tavanti A , Hensgens LAM , Ghelardi E , Campa M , Senesi S . 2007. Genotyping of Candida orthopsilosis clinical isolates by amplification fragment length polymorphism reveals genetic diversity among independent isolates and strain maintenance within patients. J Clin Microbiol 45:1455–1462. doi:10.1128/JCM.00243-07 17329454 PMC1865889

[13] Zancopé-Oliveira RM , James MJ , Derossi AP , Sampaio JL , Muniz MM , Li RK , Nascimento AS , Peralta JM , Reiss E . 2000. Strain characterization of Candida parapsilosis fungemia by molecular typing methods. Eur J Clin Microbiol Infect Dis 19:514–520. doi:10.1007/s100960000307 10968322

[14] Oliveira VKP , Paula CR , Colombo AL , Merseguel KB , Nishikaku AS , Moreira D , Ruiz L da S . 2014. Candidemia and death by Candida orthopsilosis and Candida metapsilosis in neonates and children. Pediatr Neonatol 55:75–76. doi:10.1016/j.pedneo.2013.07.006 24113226

[15] Nosek J , Tomáska L , Rycovská A , Fukuhara H . 2002. Mitochondrial telomeres as molecular markers for identification of the opportunistic yeast pathogen Candida parapsilosis. J Clin Microbiol 40:1283–1289. doi:10.1128/JCM.40.4.1283-1289.2002 11923346 PMC140342

[16] Van Asbeck EC , Clemons KV , Markham AN , Stevens DA , *Candida parapsilosis* Global Epidemiology Group . 2008. Molecular epidemiology of the global and temporal diversity of Candida parapsilosis. Scand J Infect Dis 40:827–834. doi:10.1080/00365540802144133 18609202

[17] Borman AM , Linton CJ , Oliver D , Palmer MD , Szekely A , Odds FC , Johnson EM . 2009. Pyrosequencing analysis of 20 nucleotides of internal transcribed spacer 2 discriminates Candida parapsilosis, Candida metapsilosis, and Candida orthopsilosis. J Clin Microbiol 47:2307–2310. doi:10.1128/JCM.00240-09 19403763 PMC2708508

[18] Bougnoux M-E , Tavanti A , Bouchier C , Gow NAR , Magnier A , Davidson AD , Maiden MCJ , D’Enfert C , Odds FC . 2003. Collaborative consensus for optimized multilocus sequence typing of Candida albicans. J Clin Microbiol 41:5265–5266. doi:10.1128/JCM.41.11.5265-5266.2003 14605179 PMC262540

[19] Garcia-Hermoso D , Desnos-Ollivier M , Bretagne S . 2016. Typing Candida species using microsatellite length polymorphism and multilocus sequence typing. Methods Mol Biol 1356:199–214. doi:10.1007/978-1-4939-3052-4_15 26519075

[20] Hensgens LAM , Tavanti A , Mogavero S , Ghelardi E , Senesi S . 2009. AFLP genotyping of Candida metapsilosis clinical isolates: evidence for recombination. Fungal Genet Biol 46:750–758. doi:10.1016/j.fgb.2009.06.006 19559094

[21] Asadzadeh M , Ahmad S , Hagen F , Meis JF , Al-Sweih N , Khan Z . 2015. Simple, low-cost detection of Candida parapsilosis complex isolates and molecular fingerprinting of Candida orthopsilosis strains in Kuwait by ITS region sequencing and amplified fragment length polymorphism analysis. PLoS One 10:e0142880. doi:10.1371/journal.pone.0142880 26580965 PMC4651534

[22] De Carolis E , Hensgens LAM , Vella A , Posteraro B , Sanguinetti M , Senesi S , Tavanti A . 2014. Identification and typing of the Candida parapsilosis complex: MALDI-TOF MS vs. Med Mycol 52:123–130. doi:10.1093/mmy/myt009 24577004

[23] Neji S , Trabelsi H , Hadrich I , Cheikhrouhou F , Sellami H , Makni F , Ayadi A . 2017. Molecular study of the Candida parapsilosis complex in Sfax, Tunisia. Med Mycol 55:137–144. doi:10.1093/mmy/myw063 27555560

[24] Wu Y , Zhou H , Che J , Li W , Bian F , Yu S , Zhang L , Lu J . 2014. Multilocus microsatellite markers for molecular typing of Candida tropicalis isolates. BMC Microbiol 14:245. doi:10.1186/s12866-014-0245-z 25410579 PMC4247128

[25] Gits-Muselli M , Campagne P , Desnos-Ollivier M , Le Pape P , Bretagne S , Morio F , Alanio A . 2020. Comparison of multilocus sequence typing (MLST) and microsatellite length polymorphism (MLP) for Pneumocystis Jirovecii genotyping. Comput Struct Biotechnol J 18:2890–2896. doi:10.1016/j.csbj.2020.10.005 33163149 PMC7593342

[26] Araujo R , Pina-Vaz C , Rodrigues AG , Amorim A , Gusmão L . 2009. Simple and highly discriminatory microsatellite-based multiplex PCR for Aspergillus fumigatus strain typing. Clin Microbiol Infect 15:260–266. doi:10.1111/j.1469-0691.2008.02661.x 19196262

[27] Neji S , Hadrich I , Ilahi A , Trabelsi H , Chelly H , Mahfoudh N , Cheikhrouhou F , Sellami H , Makni F , Ayadi A . 2018. Molecular genotyping of Candida parapsilosis species complex. Mycopathologia 183:765–775. doi:10.1007/s11046-018-0278-1 29995224

[28] Fan X , Xiao M , Liu P , Chen S , Kong F , Wang H , Zhang L , Hou X , Xu Y-C , Jacobsen ID . 2016. Novel polymorphic multilocus microsatellite markers to distinguish Candida tropicalis isolates. PLoS One 11:e0166156. doi:10.1371/journal.pone.0166156 27820850 PMC5098789

[29] Vatanshenassan M , Boekhout T , Mauder N , Robert V , Maier T , Meis JF , Berman J , Then E , Kostrzewa M , Hagen F . 2020. Evaluation of microsatellite typing, ITS sequencing, AFLP fingerprinting, MALDI-TOF MS, and fourier-transform infrared spectroscopy analysis of Candida auris. J Fungi (Basel) 6:146. doi:10.3390/jof6030146 32854308 PMC7576496

[30] Sabino R , Sampaio P , Rosado L , Stevens DA , Clemons KV , Pais C . 2010. New polymorphic microsatellite markers able to distinguish among Candida parapsilosis sensu stricto isolates. J Clin Microbiol 48:1677–1682. doi:10.1128/JCM.02151-09 20220157 PMC2863883

[31] Zhang L , Yu S-Y , Chen SC-A , Xiao M , Kong F , Wang H , Ning Y-T , Lu M-Y , Sun T-S , Hou X , Zhou M-L , Kang W , Zhang G , Duan S-M , Xu Y-C . 2020. Molecular characterization of Candida parapsilosis by microsatellite typing and emergence of clonal antifungal drug resistant strains in a multicenter surveillance in China. Front Microbiol 11:1320. doi:10.3389/fmicb.2020.01320 32612597 PMC7309193

[32] Leaw SN , Chang HC , Sun HF , Barton R , Bouchara JP , Chang TC . 2006. Identification of medically important yeast species by sequence analysis of the internal transcribed spacer regions. J Clin Microbiol 44:693–699. doi:10.1128/JCM.44.3.693-699.2006 16517841 PMC1393093

[33] Chen N . 2004. Using RepeatMasker to identify repetitive elements in genomic sequences. Curr Protoc Bioinformatics Chapter 4:Unit. doi:10.1002/0471250953.bi0410s05 18428725

[34] Huang JJ , Chen XF , Tsui CKM , Pang CJ , Hu ZD , Shi Y , Wang WP , Cui LY , Xiao YL , Gong J , Fan X , Li YX , Zhang G , Xiao M , Xu YC . 2022. Persistence of an epidemic cluster of Rhodotorula mucilaginosa in multiple geographic regions in China and the emergence of a 5-flucytosine resistant clone. Emerg Microbes Infect 11:1079–1089. doi:10.1080/22221751.2022.2059402 35343400 PMC9009924

[35] Singh A , Singh PK , de Groot T , Kumar A , Mathur P , Tarai B , Sachdeva N , Upadhyaya G , Sarma S , Meis JF , Chowdhary A . 2019. Emergence of clonal fluconazole-resistant Candida parapsilosis clinical isolates in a multicentre laboratory-based surveillance study in India. J Antimicrob Chemother 74:1260–1268. doi:10.1093/jac/dkz029 30753525

[36] Illnait-Zaragozí MT , Martínez-Machín GF , Fernández-Andreu CM , Perurena-Lancha MR , Theelen B , Boekhout T , Meis JF , Klaassen CH . 2012. Environmental isolation and characterisation of Cryptococcus species from living trees in Havana city, Cuba. Mycoses 55:e138–44. doi:10.1111/j.1439-0507.2012.02168.x 22364253

[37] Hunter PR , Gaston MA . 1988. Numerical index of the discriminatory ability of typing systems: an application of simpson’s index of diversity. J Clin Microbiol 26:2465–2466. doi:10.1128/jcm.26.11.2465-2466.1988 3069867 PMC266921

[38] Gosselin S , Fullmer MS , Feng Y , Gogarten JP . 2022. Improving phylogenies based on average nucleotide identity incorporating saturation correction and nonparametric bootstrap support. Syst Biol 71:396–409. doi:10.1093/sysbio/syab060 34289044 PMC8830074

[39] Organization WH . 2022. WHO fungal priority pathogens list to guide research, development and public health action. Available from: https://www.who.int/publications/i/item/9789240060241. Retrieved 25 Oct 2022.

[40] Merseguel KB , Nishikaku AS , Rodrigues AM , Padovan AC , e Ferreira RC , de Azevedo Melo AS , Briones MR da S , Colombo AL . 2015. Genetic diversity of medically important and emerging Candida species causing invasive infection. BMC Infect Dis 15:57. doi:10.1186/s12879-015-0793-3 25887032 PMC4339437

[41] Tay ST , Na SL , Chong J . 2009. Molecular differentiation and antifungal susceptibilities of Candida parapsilosis isolated from patients with bloodstream infections. J Med Microbiol 58:185–191. doi:10.1099/jmm.0.004242-0 19141735

[42] Amouri I , Sellami H , Abbes S , Hadrich I , Mahfoudh N , Makni H , Ayadi A . 2012. Microsatellite analysis of Candida isolates from recurrent vulvovaginal Candidiasis. J Med Microbiol 61:1091–1096. doi:10.1099/jmm.0.043992-0 22538998

[43] Barbedo LS , Vaz C , Pais C , Figueiredo-Carvalho MHG , Muniz M de M , Zancope-Oliveira RM , Sampaio P . 2015. Different scenarios for Candida parapsilosis fungaemia reveal high numbers of mixed C. Parapsilosis and Candida orthopsilosis infections. J Med Microbiol 64:7–17. doi:10.1099/jmm.0.080655-0 25351711

