# Supplementary material for: The first established microsatellite markers to distinguish Candida orthopsilosis isolates and detection of a nosocomial outbreak in China
Source: J Clin Microbiol. 2023 Oct 25;61(11):e00806-23. doi: 10.1128/jcm.00806-23 (PMC10662339; doi:10.1128/jcm.00806-23)
Supplement: Table S1 — The fragment size of four loci of 48 samples and ATCC96139. [file jcm.00806-23-s0001.docx]

| Table S1: the fragment size of 48 samples and ATCC96139 using four loci to detect | | | | | | | | | | | | |
| --- | --- | --- | --- | --- | --- | --- | --- | --- | --- | --- | --- | --- |
| COMT TYPE | SAMPLE | Hospital | Biological sample | Date of isolation | CO4 | | CO11 | | CO32 | | CO49 | |
| COMT1 | ATCC96139 | / | / | / | 249 | 282 | 267 | 303 | 250 | 274 | 243 | 261 |
| COMT2 | 2563 | H10 | Blood | 4/28/2018 | 249 | 249 | 267 | 267 | 252 | 252 | 249 | 249 |
| COMT3 | 75 | H01 | Blood | 11/3/2017 | 249 | 249 | 267 | 267 | 252 | 252 | 255 | 255 |
| COMT4 | 4249 | H22 | Ascites | 10/20/2018 | 252 | 252 | 276 | 276 | 252 | 252 | 246 | 246 |
| COMT5 | 757 | H09 | Catheter | 8/19/2017 | 252 | 252 | 276 | 276 | 274 | 274 | 246 | 246 |
| COMT5 | 2130 | H04 | Tissue | 7/9/2018 | 252 | 252 | 276 | 276 | 274 | 274 | 246 | 246 |
| COMT5 | 2143 | H09 | Catheter | 7/1/2018 | 252 | 252 | 276 | 276 | 274 | 274 | 246 | 246 |
| COMT5 | 2190 | H09 | Blood | 6/14/2019 | 252 | 252 | 276 | 276 | 274 | 274 | 246 | 246 |
| COMT5 | 2849 | H09 | Blood | 7/6/2019 | 252 | 252 | 276 | 276 | 274 | 274 | 246 | 246 |
| COMT5 | 3933 | H09 | Catheter | 9/9/2017 | 252 | 252 | 276 | 276 | 274 | 274 | 246 | 246 |
| COMT5 | CH616 | H09 | Blood | 6/15/2019 | 252 | 252 | 276 | 276 | 274 | 274 | 246 | 246 |
| COMT5 | CH618 | H09 | Catheter | 7/19/2019 | 252 | 252 | 276 | 276 | 274 | 274 | 246 | 246 |
| COMT5 | CH624 | H09 | catheter | 6/7/2018 | 252 | 252 | 276 | 276 | 274 | 274 | 246 | 246 |
| COMT5 | CH628 | H13 | Blood | 11/30/2018 | 252 | 252 | 276 | 276 | 274 | 274 | 246 | 246 |
| COMT5 | CH630 | H09 | Blood | 6/22/2019 | 252 | 252 | 276 | 276 | 274 | 274 | 246 | 246 |
| COMT5 | CH635 | H09 | Blood | 7/16/2019 | 252 | 252 | 276 | 276 | 274 | 274 | 246 | 246 |
| COMT5 | CH641 | H21 | Catheter | 6/27/2018 | 252 | 252 | 276 | 276 | 274 | 274 | 246 | 246 |
| COMT6 | 594 | H06 | Ascites | 4/9/2018 | 264 | 264 | 276 | 276 | 258 | 258 | 243 | 243 |
| COMT6 | 1424 | H06 | Ascites | 4/9/2018 | 264 | 264 | 276 | 276 | 258 | 258 | 243 | 243 |
| COMT6 | 1425 | H03 | Blood | 2/21/2018 | 264 | 264 | 276 | 276 | 258 | 258 | 243 | 243 |
| COMT7 | 1440 | H06 | Pus | 6/5/2018 | 267 | 267 | 261 | 261 | 262 | 262 | 249 | 249 |
| COMT7 | 2499 | H10 | Bile | 3/22/2018 | 267 | 267 | 261 | 261 | 262 | 262 | 249 | 249 |
| COMT7 | 3161 | H17 | Blood | 7/16/2018 | 267 | 267 | 261 | 261 | 262 | 262 | 249 | 249 |
| COMT8 | 1693 | H07 | Catheter | 11/14/2018 | 267 | 267 | 261 | 261 | 252 | 252 | 249 | 249 |
| COMT8 | 3240 | H18 | Blood | 10/25/2018 | 267 | 267 | 261 | 261 | 252 | 252 | 249 | 249 |
| COMT9 | 3034 | H16 | Blood | 8/29/2018 | 267 | 267 | 261 | 261 | 252 | 262 | 249 | 249 |
| COMT10 | 618 | H17 | Blood | 6/7/2018 | 282 | 282 | 300 | 300 | 250 | 274 | 243 | 261 |
| COMT11 | 3145 | H03 | Blood | 6/6/2018 | 282 | 282 | 300 | 300 | 274 | 274 | 243 | 261 |
| COMT12 | 475 | H05 | Pus | 12/5/2017 | 249 | 249 | 264 | 264 | 252 | 252 | 249 | 249 |
| COMT12 | 489 | H19 | Blood | 5/17/2018 | 249 | 249 | 264 | 264 | 252 | 252 | 249 | 249 |
| COMT12 | 1327 | H08 | Catheter | 8/21/2018 | 249 | 249 | 264 | 264 | 252 | 252 | 249 | 249 |
| COMT12 | 1917 | H08 | Blood | 11/24/2018 | 249 | 249 | 264 | 264 | 252 | 252 | 249 | 249 |
| COMT12 | 1987 | H11 | Catheter | 11/13/2017 | 249 | 249 | 264 | 264 | 252 | 252 | 249 | 249 |
| COMT12 | 2194 | H12 | Blood | 11/20/2017 | 249 | 249 | 264 | 264 | 252 | 252 | 249 | 249 |
| COMT12 | 2719 | H15 | Catheter | 11/5/2018 | 249 | 249 | 264 | 264 | 252 | 252 | 249 | 249 |
| COMT12 | 2723 | H23 | Blood | 6/23/2018 | 249 | 249 | 264 | 264 | 252 | 252 | 249 | 249 |
| COMT12 | 2805 | H18 | Blood | 6/24/2018 | 249 | 249 | 264 | 264 | 252 | 252 | 249 | 249 |
| COMT12 | 2859 | H14 | Blood | 1/13/2018 | 249 | 249 | 264 | 264 | 252 | 252 | 249 | 249 |
| COMT12 | 2972 | H18 | Blood | 4/6/2018 | 249 | 249 | 264 | 264 | 252 | 252 | 249 | 249 |
| COMT12 | 3189 | H14 | Blood | 9/26/2017 | 249 | 249 | 264 | 264 | 252 | 252 | 249 | 249 |
| COMT12 | 3203 | H09 | Ascites | 12/11/2019 | 249 | 249 | 264 | 264 | 252 | 252 | 249 | 249 |
| COMT12 | 3204 | H12 | Blood | 12/7/2018 | 249 | 249 | 264 | 264 | 252 | 252 | 249 | 249 |
| COMT12 | 3371 | H09 | Ascites | 6/24/2018 | 249 | 249 | 264 | 264 | 252 | 252 | 249 | 249 |
| COMT12 | 3372 | H18 | Blood | 6/24/2018 | 249 | 249 | 264 | 264 | 252 | 252 | 249 | 249 |
| COMT12 | 3633 | H19 | Tissue | 5/15/2018 | 249 | 249 | 264 | 264 | 252 | 252 | 249 | 249 |
| COMT12 | 3899 | H20 | Blood | 9/30/2018 | 249 | 249 | 264 | 264 | 252 | 252 | 249 | 249 |
| COMT12 | 3985 | H11 | Blood | 11/19/2017 | 249 | 249 | 264 | 264 | 252 | 252 | 249 | 249 |
| COMT12 | 4347 | H02 | Blood | 7/23/2018 | 249 | 249 | 264 | 264 | 252 | 252 | 249 | 249 |
| COMT12 | CH670 | H02 | Blood | 7/2/2018 | 249 | 249 | 264 | 264 | 252 | 252 | 249 | 249 |
